# Protective Effect of Safranal, a Constituent of *Crocus sativus, *on Quinolinic Acid-induced Oxidative Damage in Rat Hippocampus

**Published:** 2013-01

**Authors:** Hamid Reza Sadeghnia, Mina Kamkar, Elham Assadpour, Mohammad Taher Boroushaki, Ahmad Ghorbani

**Affiliations:** 1 Department of Pharmacology, School of Medicine, Mashhad University of Medical Sciences, Mashhad, Iran; 2 Pharmacological Research Center of Medicinal Plants, School of Medicine, Mashhad University of Medical Sciences, Mashhad, Iran; 3 Neuroscience Research Center, Department of New Sciences and Technology, School of Medicine, Mashhad University of Medical Sciences, Mashhad, Iran

**Keywords:** Crocus sativus, Hippocampus, Neurodegenerative disorders, Oxidative stress, Quinolinic acid, Safranal

## Abstract

***Objective(s): ***Quinolinic acid (QA)-mediated excitotoxicity has been widely used as a model for studying neurodegenerative disorders. Recent studies suggested that saffron (*Crocus sativus*) or its active metabolite, i.e. safranal, exerts pharmacological actions on central nervous system including anxiolytic, anticonvulsant, and neuroprotective properties. The present study aimed to investigate the effect safranal pretreatment on QA-induced oxidative damage in rat hippocampus.

***Materials and Methods: ***Under anesthesia, a guide cannula was stereotaxically inserted into left ventral hippocampus of rats. The rats were then given either saline or safranal (72.75, 145.5, and 291 mg/kg, IP) 30 min before administration of QA (300 nmol, intrahippocampal injection). The markers of oxidative stress including thiobarbituric acid reactive substances (TBARS, as an index of lipid preoxidation), total sulfhydryl groups, antioxidant capacity of hippocampus (using FRAP assay), and oxidative DNA damage (%tail DNA, using comet assay) were measured in hippocampus.

***Results:*** The QA induced a significant increase in TBARS levels and %tail DNA and remarkable decrease in antioxidant power (FRAP value) and total sulfhydryl content of hippocampus, in comparison with control animals. Systemic administration of safranal (291 mg/kg, IP), effectively and dose-dependently decreased the QA-induced lipid peroxidation (*P*<0.001) and oxidative DNA damage (*P*<0.001). Safranal also prevented the decrease of hippocampal thiol redox and antioxidant status (*P*<0.001) produced by QA.

***Conclusion:*** Safranal have protective effects on different markers of oxidative damage in hippocampal tissue following QA administration. Our findings might raise a possibility of potential therapeutic application of safranal for preventing and treating neurodegenerative disorders such as Alzheimer’s disease.

## Introduction

A growing body of evidence suggests that excessive activation of glutamate receptors and subsequent excitotoxicity and oxidative stress are involved in the pathophysiology of many neurological and neurodegenerative disorders (1, 2). Quinolinic acid (2,3-pyridine dicarboxylic acid, QA), a major tryptophan metabolite produced in the kynurenine pathway (KP), is an endogenous agonist at receptors for the glutamate analogue, N-methyl-D-aspartate (NMDA), and has been hypothetically linked to the pathogenesis of a variety of neurodegenerative as well as inﬂammatory and non-inﬂammatory human neurological and psychological diseases such as schizophrenia or HIV-associated dementia (HAD) (3-6). Under brain inflammatory conditions, QA is produced by activated microglia and infiltrating macrophages which leads to neurotoxicity (7). A diversity of toxic mechanisms have been proposed for QA including NMDA receptors overactivation and increased intracellular Ca^2+^ concentrations followed by mitochondrial dysfunction and cytochrome c release, decreased ATP production, formation of damaging free radicals, i.e., reactive oxygen, and nitrogen species (ROS and RNS, respectively) which in turn leads to oxidative stress and neuroinflammation (8,9). Furthermore, QA increases glutamate release and decreases glutamate uptake, *in vivo* and *in vitro* (10,11). Therefore, QA-mediated excitotoxicity has been widely used as a model for studying neurodegenerative disorders and also to investigate possible pharmacological interventions against excitotoxic neuronal damage (12, 13). 

*Crocus sativus* (saffron), a perennial stemless herb of the Iridaceae family, is widely cultivated in Iran and also in some other countries such as India. Safranal (2,6,6-trimethyl-1,3- cyclohexadiene-1-carboxaldehyde, C_10_H_14_O) is the main constituent of *C. sativus* essential volatile oil (14). Recently, numerous neuropharmacological properties such as antioxidant, analgesic and anti-inflammatory, hypnotic and anxiolytic, anticonvulsant, anti-ischemic, antidepressant, and neuroprotective have been reported for safranal (15-24). In a recent study, reduction of metabolic and behavioral signs of acute stress in rats by saffron extract has been attributed to its constituent, safranal (25). 

Recently, subacute (21 days) and acute (2 days) toxicity of safranal were determined in mice and rats. In rats, administration of safranal with dose of 425 mg/kg/day (0.5 ml/kg/day) once-daily for 21 days, caused a significant decrease in food and water consumption and body weight while an increase in serum levels of lactic acid dehydrogenase and urea nitrogen was observed. Pathological data also showed some toxic effects in kidneys and lungs. In acute toxicity test, the LD50 (50% lethal dose) values of safranal were found to be 1260 mg/kg (1.48 ml/kg) and 1275 mg/kg (1.50 ml/kg) in male mice and rats, respectively (26). 

Considering the possible involvement of QA in the neuropathogenesis of several major neurological diseases such as Alzheimer’s disease, Huntington's disease, epilepsy, and postischemic neuronal damage, the present study focused on the possible protective effects of safranal against oxidative damages induced by intrahippocampal injection of quinolinic acid.

## Materials and Methods

***Chemicals***

Safranal and quinolinic acid (QA) were purchased from Fluka (St. Gallen, Switzerland) and Sigma (St. Louis, US), respectively. DTNB (2,2'-dinitro-5, 5'-dithiodibenzoic acid), tripyridyltriazine (TPTZ), TBA (2-thiobarbituric acid), Tris (hydroxymethyl) aminomethane (Trizma base), ethylene diamine tetraacetic acid disodium salt (Na_2_EDTA), t-octylphenoxypoly-ethoxyethanol (Triton X-100), sodium lauroyl sarcosinate (sarkosyl), ethidium bromide, methanol, sodium acetate, glacial acetic acid, phosphoric acid, potassium chloride, ferric chloride, ferrous sulfate, chloral hydrate, and hydrochloric acid were obtained from Merck (Dramstadt, Germany). Low melting point (LMP) and normal melting point (NMP) agarose were purchased from Biogen (Mashhad, Iran) and Fermentase (Glen Burnie, US), respectively.

***Animals***

Adult male Wistar rats weighting 250-300 g from the Central Animal House of Mashhad University of Medical Sciences (Mashhad, Iran), were used throughout the study. The animals were housed in the same room under a constant temperature (22±2 °C) and standard conditions of a 12h light/dark cycle with free access to food pellets and tap water, available *ad libitum*. The experimental protocol was approved by the Animal Care and Use Committee (87534), Mashhad University of Medical Sciences and was performed in accordance with the National Institutes of Health Guidelines for the Care and Use of Laboratory Animals.

***Treatment schedule***

The animals were randomly divided into five different experimental groups of seven animals each. Group 1 (sham group) received single intraperitoneal (IP) injection of normal saline (10 ml/kg) plus 1 µl of normal saline which was infused into the left hippocampus, 30 min later. Group 2 (QA group) received single IP injection of normal saline (10 ml/kg) plus intrahippocampal (IH) administration of QA (300 nmol/1 μl/rat), 30 min later. Groups 3-5 (treatment groups) were injected by safranal (72.75, 145.5, and 291 mg/kg, IP), 30 min prior to QA administration (300 nmol/1 μl/rat, IH). 

***Intrahippocampal administration of QA***

**Figure 1:**
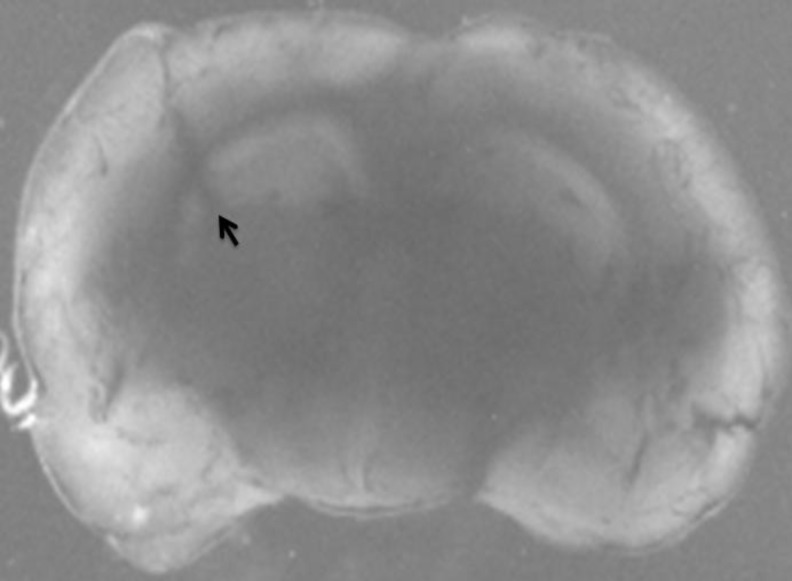
Photograph representing the microinjection site of quinolinic acid into the ventral hippocampus (black arrow)

The animals were anesthetized with chloral hydrate (400 mg/kg, IP and then positioned in a stereotaxic apparatus (Stoelting, US). After exposing the bregma suture, a small burr hole was made through the skull to permit access of microinjection needle into the left ventral hippocampus according to the brain atlas of Paxinos and Watson (AP 3.7 mm, ML 2.4 mm, and DV 3.2 mm) (27). Using a 29-gauge stainless steel needle connected to a Hamilton syringe (Bonaduz, GR, Switzerland), one microliter saline solution containing 300 nmol QA (or vehicle alone as control) was unilaterally microinjected into the left ventral hippocampus region over a period of 1 min and left *in situ* for another 1 min to prevent back diffusion of the injected drug solution (28, Figure 1). Following surgery, the animals were kept warm to recover from surgery and maintained in suitable situation for 24 hr. After that, the animals were decapitated, brains were quickly removed, kept in ice-cold saline, and the extracted hippocampi were immediately frozen in liquid nitrogen and maintained at -80°C until processing. The injection site was also verified using 1 µl methylene blue and anatomical observation. 

The left hippocampus portion was gently homogenized in ice-cold phosphate buffered saline (0.1 M, pH 7.4) to give a 10% homogeny suspension and used for biochemical and comet assay.

***Ferric reducing/antioxidant power (FRAP) assay***

The basis of FRAP assay is reducing the colorless Fe^III^-TPTZ complex to blue colored Fe^II^-TPTZ complex, by action of electron donating antioxidants in biological samples (29). The FRAP reagent consists of 300 mM acetate buffer (pH=3.6), 10 mM TPTZ in 40 mM HCl, and 20 mM FeCl_3_.6H_2_O in the ratio of 10:1:1.

Briefly, 50 μl of homogenate was added to 1.5 ml freshly prepared and prewarmed (37ºC) FRAP reagent in a test tube and incubated at 37ºC for 10 min. The absorbance of the blue colored complex was read against reagent blank (1.5 ml FRAP reagent + 50 μl distilled water) at 593 nm. Standard solutions of Fe^II^ in the range of 100 to 1000 mM were prepared from ferrous sulphate (FeSO_4_.7H_2_O) in distilled water. FRAP values were expressed as nmol ferric ions reduced to ferrous form/mg tissue (29).

***Total sulfhydryl (SH) groups measurement***

Total thiol content was estimated based on the Ellman method (30). In this method, SH groups react with chromogenic DTNB and produce a yellow-colored dianion (5-thio-2- nitrobenzoic acid, TNB), which has peak absorbance at 412 nm. 

Briefly, 1 ml Tris-EDTA buffer (0.1 M Tris, 10 mM EDTA, pH=8.6) was added to 50 µl homogenate sample in 2 ml cuvettes. Sample absorbance was read at 412 nm against Tris-EDTA buffer alone (A_1_), then 20 µl DTNB reagent (10 mM in methanol) was added to the mixture. Following 15 min incubation at room temperature, the sample absorbance was read again (A_2_). DTNB reagent absorbance was also read as a blank (B). Total thiol concentration was calculated by the following equation and expressed as nmol/mg tissue (22).

Total thiol concentration (mM) = (A_2_-A_1_-B) × (1.07/0.05) × 13.6

***Thiobarbituric acid reactive species measurement***

Hippocampal lipid peroxides formation was measured as malondialdehyde (MDA), which is the end product of lipid peroxidation and reacts with thiobarbituric acid (TBA) as a TBA reactive substance (TBARS) to produce a pink colored complex which has peak absorbance at 535 nm (31). In brief, 1 ml of homogenate sample was mixed with 2 ml of TCA-TBA-HCl reagent (15% TCA, 0.67% TBA, and 0.25N HCl) and heated for 45 min in a boiling water bath. After cooling, the mixture was centrifuged at 3000 rpm for 10 min. The supernatant was collected, and the absorbance was read against blank, at 535 nm. The amount of MDA produced was calculated, using a molar absorption coefficient of 1.56×10^5 ^M^-1^cm^-1^ and expressed as nmol/g tissue (32).

***Alkaline single cell gel electrophoresis (SCGE) assay***

The in vivo alkaline SCGE (comet) assay was conducted based on the method described by Sasaki *et al* with some modifications (33). In brief, 10 µl of the hippocampus cells suspension, prepared as above, was mixed with 90 µl LMP agarose (0.5% in physiological saline), and the mixture was quickly layered over a microscope slide precoated with a layer of 100 µl NMP agarose (1% in physiological saline), the slides were then covered with a cover slip, and placed on ice to allow agarose to gel. Finally, another layer of LMP agarose was added on top. The slides were immersed immediately in a chilled lysing solution (pH 10) made up of 2.5 M NaCl, 100 mM Na_2_EDTA, 10 mM Trizma, 1% sarkosyl, 10% DMSO, and 1% Triton X-100, and kept at 0^○^C in the dark overnight. Then, the slides were placed on a horizontal gel electrophoresis platform and covered with a chilled alkaline solution made up of 300 mM NaOH and 1 mM Na_2_EDTA (pH>13). They were left in the solution in the dark at 0^○^C for 40 min, and then electrophoresed at 0^○^C in the dark for 30 min at 25 V and approximately 300 mA. The slides were rinsed gently three times with 400 mM Trizma solution (pH 7.5) to neutralize the excess alkali, stained with 50 µl of 20 mg/mL ethidium bromide, and covered with a cover slip.

One hundred nuclei per organ from each animal (50 nuclei on one slide) were examined and photographed using a fluorescence microscope (Nikon, Kyoto, Japan) at 400X magnification equipped with an excitation filter of 520-550 nm and a barrier filter of 580 nm. Undamaged cells resemble an intact nucleus without a tail, and damaged cells have the appearance of a comet. The percent of DNA in the comet tail (%tail DNA), which is an estimate of DNA damage, was measured using a computerized image analysis software (CASP software). 

***Statistical analysis***

The statistical analysis was performed using Prism 5.00 for Windows software (Graph-Pad Software, San Diego, CA). Data were expressed as mean±SEM. Comparisons between the study groups were made using one-way ANOVA followed by Tukey-Kramer post-hoc test for multiple comparisons. The p-values less than 0.05 were considered to be statistically significant.

## Results

***Effect of safranal on lipid peroxidation level***

**Figure 2:**
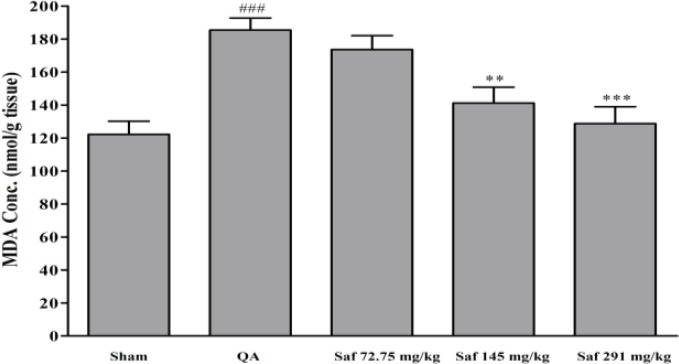
Effect of safranal on malondialdehyde (MDA) level of hippocampus homogenate samples following microinjection of quinolinic acid (QA, 300 nmol) into rat hippocampus. Values are mean±SEM (n=8). ^**^P<0.01, ^***^P<0.001 as compared with QA-treated animals; ^###^*P*<0.001 as compared with saline-treated animals (One-way ANOVA followed by Tukey-Kramer test)

The amount of QA-induced free radical damage was assessed using lipid peroxidation, which was measured as MDA levels. There was a significant increase (51.7%) in the MDA levels following QA administration as compared with sham-operated animals (122.2±7.9 vs. 185.4±7.3 nmol/g tissue, *P*<0.001) (Figure 2). Safranal pretreatment resulted in a significant and dose-dependent reduction in the levels of MDA. In safranal-pretreated groups with doses of 72.75 mg/kg, 145.5 mg/kg, and 291 mg/kg, MDA levels were 173.6, 141.2, and 128.7 nmol/g tissue, respectively (Figure 2).

***Effect of safranal on FRAP value***

**Figure 3:**
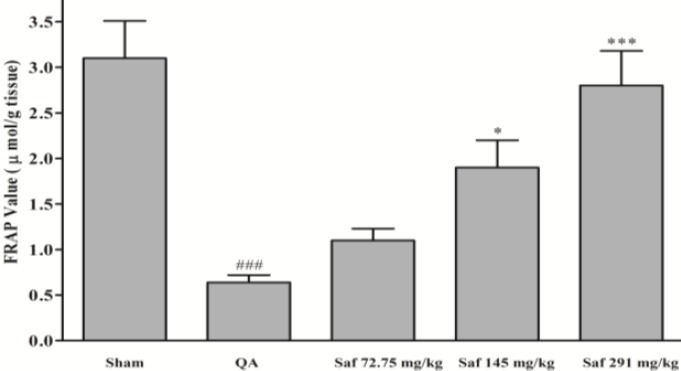
Effect of safranal on antioxidant power (FRAP value) of hippocampus homogenate samples following microinjection of quinolinic acid (QA, 300 nmol) into rat hippocampus. Values are mean±SEM (n=8). ^*^P<0.01, ^***^ P<0.001 as compared with QA-treated animals; ^###^*P*<0.001 as compared with saline-treated animals (One-way ANOVA followed by Tukey-Kramer test)

QA caused a significant reduction in FRAP value (79.3%) of homogenate samples as compared with sham-operated animals (3.1±0.4 vs. 0.64±0.08 μmol/g tissue, *P*<0.001) (Figure 3). Safranal pretreatment increased antioxidant power (FRAP value) of brain homogenate samples, in a dose-dependent manner (from 0.64±0.08 to 2.8±0.4 μmol/g tissue, *P*<0.001, 291 mg/kg) (Figure 3).

***Effect safranal on total thiol content***

**Figure 4:**
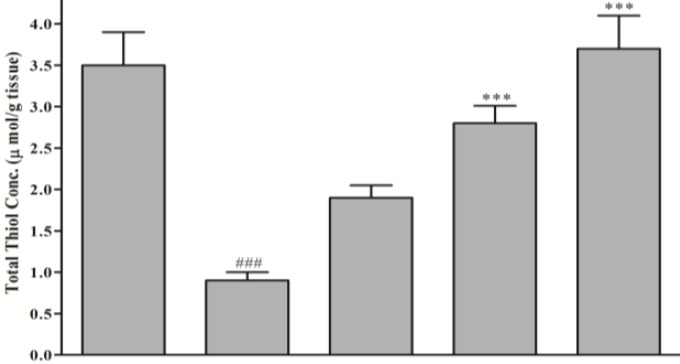
Effect of safranal on total thiol concentration of hippocampus homogenate samples following microinjection of quinolinic acid (QA, 300 nmol) into rat hippocampus. Values are mean±SEM (n=8). ^***^ P<0.001 as compared with QA-treated animals; ^###^ *P*<0.001 as compared with saline-treated animals (One-way ANOVA followed by Tukey-Kramer test)

Following QA microinjection a significant reduction (74.3%) in total SH content (3.5±0.4 vs. 0.9±0.1 μmol/g tissue, *P*<0.001) was observed (Figure 4). Safranal pretreatment produced significant and dose-dependent elevation in total thiol concentration, as compared with QA group (from 0.9±0.1 to 3.7±0.40 μmol/g tissue, *P*<0.001, 291 mg/kg) (Figure 4).

***Effect of safranal on DNA damage***

**Figure 5:**
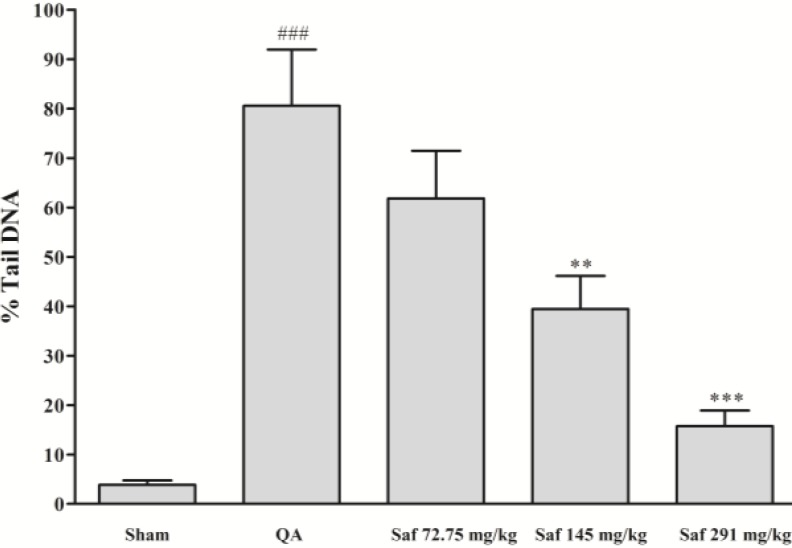
Effect of safranal on DNA damage (percent of DNA in the comet tail, %tail DNA) following microinjection of quinolinic acid (QA, 300 nmol) into rat hippocampus. Values are mean±SEM (n=8). ^**^P<0.01, ^***^P<0.001 as compared with QA-treated animals; ^###^* P*<0.001 as compared with saline-treated animals (One-way ANOVA followed by Tukey-Kramer test)

As shown in Figure 4, a significant increase in the %tail DNA was seen in hippocampal nuclei of QA-treated rats, as compared with those of sham group (*P*<0.001). In contrast, safranal significantly decreased DNA damage induced by QA, in a dose-dependent manner (Figure 5).

## Discussion

Therapeutic options for neurodegenerative disorders such as stroke, Huntington’s disease, Parkinson’s disease, and Alzheimer’s disease are limited and inadequate to control these diseases. Therefore, the search for new effective natural products with neuroprotective properties is a promising approach for drug discovery and development (34). 

In this study, we found that QA caused significant hippocampal neuronal lesion and oxidative stress marker by increasing lipid peroxidation, oxidative DNA damage, and depletion of sulfhydryl groups and antioxidant power in the rat hippocampus, as previously reported in the literature (35-37). In addition, we described for the first time some protective effects of safranal against QA-induced oxidative damage in the rat hippocampus. We found that safranal exhibits no toxic effects on hippocampus at high concentration used alone (291 mg/kg, data not shown). Free radical-induced DNA damage is an important cause of numerous diseases (38). Our results showed that pretreatment with safranal significantly and dose-dependently decreased free radical-mediated lipid peroxidation and DNA damage and improved hippocampal antioxidant status following QA insult. 

The present results demonstrated that safranal at low dose (72.75 mg/kg) was not able to reduce QA-induced oxidative damage, but when administered at relatively high concentration is possible to obtain inhibition of lipid peroxidation, restore thiol redox and antioxidant status and decrease DNA damage. While QA increased the percent of DNA in the comet tail (%tail DNA) from 3.84% (in sham group) to 80.56%, pretreatment with safranal (291 mg/kg) significantly decreased QA-induced DNA damage to about 15.73%. 

Excitotoxicity and resultant oxidative stress is a major pathological mechanism involved in neuronal degeneration and many central nervous system (CNS) disorders (39). In inflammatory conditions, oxidative tryptophan metabolism via kynurenine pathway is often routed preferentially towards the production of QA (7). Brain structures, especially hippocampus, are very sensitive to the exposure to high concentration of QA which causes significant neurodegeneration (40). QA has been reported to be associated with several brain pathologies including neurological and neurodegenerative disorders (Alzheimer’s disease, Parkinson’s disease, Huntington’s disease, and epilepsy) and affective disorders (HIV-assocaited dementia, schizophrenia, depression, and anxiety) (4,7). It is now widely accepted that QA-induced neurotoxicity is mediated through several mechanisms including excessive NMDA receptor activation leading to massive increase in intracellular Ca^2+ ^and causing oxidative damage as a result of massive free radicals formation. In addition, QA overstimulates the glutamatergic system by inhibition of glutamate uptake and stimulation of glutamate release (10). QA is also pro-oxidant in the nature and can generate reactive oxygen species by a receptor-independent mechanism through chelate formation with ferrous ion (41). It is well documented that the nervous system is especially vulnerable to free radical-mediated injury due to several reasons including high oxygen consumption, high contents of polyunsaturated fatty acids (PUFA) which are particularly vulnerable to free radical attack, relatively low antioxidant defense mechanisms, and high Ca^2+^ trafficking across neuronal membranes (42).

Considering the role of reactive oxygen species in QA-induced neuronal death, safranal has been reported to exhibit antioxidant and free radical scavenger properties (15). Recently, an inhibitory effect of safranal on deoxyribose oxidation, erythrocyte membrane, and liver microsomal non-enzymatic lipid peroxidation has been described by Hosseinzadeh *et al* (19). The protective effect of safranal against subacute toxicity of diazinon has also been reported mediated through its antioxidant properties (43). In a rat model of global cerebral ischemia, safranal restored some biomarkers of oxidative stress (21). In support of this view, Bharti *et al* showed that safranal dose-dependently normalized myocardial antioxidant levels, cardiac injury markers (lactic acid dehydrogenase and CK-MB), and decreased TNF-α level in ischemia-reperfusion-insulted rats myocardium, probably due to its fortified antioxidant and anti-apoptotic potential (44). 

Recently, it has been shown that safranal could inhibit kainic acid-evoked glutamate release in rat hippocampus (45). Based on this information and the fact that some neurotoxic actions of QA are also related to disturbances on glutamate release and uptake, we can hypothesize that safranal by decreasing synaptic glutamate concentration and reduced QA-induced oxidative damage. 

Excitotoxicity produced by QA was also associated with gamma-aminobutyric acid (GABA) depletion and specific GABAergic neuronal death (5, 46, 47). Moreover, it has been shown that following QA lesion of rat striatum, there was a marked increase in the central and peripheral GABA_A_/benzodiazepine receptors (46). Furthermore, the peripheral benzodiazepine receptor ligand, PK11195, has been shown to reduce microglial activation, inflammatory responses, oxidative damage, and neuronal death in QA-injected rat striatum (48). It has also been reported that diazepam, a well-known benzodiazepine agonist, is anticonvulsant against QA-induced seizures (49). Several studies have proposed that safranal exerts its anticonvulsant effects by acting on GABA_A_/benzodiazepine receptor complex which may play a role in protecting against QA insult (22, 50). 

Neuroinflammation is one of the key features of acute and chronic neurodegenerative conditions (51, 52). Several studies have shown that QA causes remarkable upregulation of proinﬂammatory cytokines (IL-1β, IL-6 and TNF-α) and activation of caspase 3, leading to apoptosis of neurons and glial cells, *in vitro* and *in vivo* (36, 48, 53). Recently, strong anti-inflammatory and anti-apoptotic potential have been described for safranal. These effects have been shown to mediate through direct suppression of IKK-/NF-κB/Bax/caspase3/TNF-α or upregulation of Bcl2 expression (44). Therefore, inhibition of inflammatory responses may be another plausible mechanism for alleviating effects of safranal against QA-induced lesion.

## Conclusions

The present study demonstrated that safranal has protective effects on QA-induced oxidative damage in rat hippocampus through inhibition of lipid peroxidation and oxidative DNA damage and improving hippocampal antioxidant and thiol redox status, suggesting its antioxidant and neuroprotective properties.

## References

[1] Gilgun-Sherki Y, Melamed E, Offen D (2001). Oxidative stress induced-neurodegenerative diseases: the need for antioxidants that penetrate the blood brain barrier. Neuropharmacol.

[2] Lau A, Tymianski M (2010). Glutamate receptors, neurotoxicity and neurodegeneration. Eur J Physiol.

[3] Heyes MP, Saito K, Crowley JS, Davis LE, Demitrack MA, Der M (1992). Quinolinic acid and kynurenine pathway metabolism in inflammatory and non-inflammatory neurological disease. Brain.

[4] Myint AM (2012). Kynurenines: from the perspective of major psychiatric disorders. FEBS J.

[5] Stone TW (1993). Neuropharmacology of quinolinic and kynurenic acids. Pharmacol Rev.

[6] Zadori D, Klivenyi P, Vamos E, Fulop F, Toldi J, Vecsei L (2009). Kynurenines in chronic neurodegenerative disorders: future therapeutic strategies. J Neural Transm.

[7] Chen Y, Guillemin GJ (2009). Kynurenine pathway metabolites in humans: disease and healthy states. Int J Tryptophan Res.

[8] Pérez-De LaCruzV, Carrillo-Mora P, Santamaría A (2012). Quinolinic Acid, an endogenous molecule combining excitotoxicity, oxidative stress and other toxic mechanisms. Int J Tryptophan Res.

[9] Sas K, Robotka H, Toldi J, Vécsei L (2007). Mitochondria, metabolic disturbances, oxidative stress and the kynurenine system, with focus on neurodegenerative disorders. J Neurol Sci.

[10] Tavares RG, Tasca CI, Santos CE, Alves LB, Porciúncula LO, Emanuelli T (2002). Quinolinic acid stimulates synaptosomal glutamate release and inhibits glutamate uptake into astrocytes. Neurochem Int.

[11] Tavares RG, Schmidt AP, Abud J, Tasca CI, Souza DO (2005). In vivo quinolinic acid increases synaptosomal glutamate release in rats: reversal by guanosine. Neurochem Res.

[12] Vezzani A, Forloni GL, Serafini R, Rizzi M, Samanin R (1991). Neurodegenerative effects induced by chronic infusion of quinolinic acid in rat striatum and hippocampus. Eur J Neurosci.

[13] Dairam A, Chetty P, Daya S (2006). Non-steroidal anti-inflammatory agents, tolmetin and sulindac, attenuate oxidative stress in rat brain homogenate and reduce quinolinic acid-induced neurodegeneration in rat hippocampal neurons. Metab Brain Dis.

[14] Tarantilis PA, Tsoupras G, Polissiou M (1995). Determination of saffron (Crocus sativus L) components in crude plant extract using high performance liquid chromatography- UV-visible photodiode-array detection-mass spectrometry. J Chromatogr A.

[15] Assimopoulou AN, Sinakos Z, Papageorgiou VP (2005). Radical scavenging activity of Crocus sativus L extract and its bioactive constituents. Phytother Res.

[16] Amin B, Hosseinzadeh H (2012). Evaluation of aqueous and ethanolic extracts of saffron, Crocus sativus L and its constituents, safranal and crocin in allodynia and hyperalgesia induced by chronic constriction injury model of neuropathic pain in rats. Fitoterapia.

[17] Hosseinzadeh H, Shariaty V (2007). Anti-nociceptive effect of safranal, a constituent of Crocus sativus (saffron), in mice. Pharmacologyonline.

[18] Fukui H, Toyoshima K, Komaki R (2011). Psychological and neuroendocrinological effects of odor of saffron (Crocus sativus). Phytomedicine.

[19] Hosseinzadeh H, Noraei NB (2009). Anxiolytic and hypnotic effect of Crocus sativus aqueous extract and its constituents, crocin and safranal, in mice. Phytother Res.

[20] Liu Z, Xu XH, Liu TY, Hong ZY, Urade Y, Huang ZL (2012). Safranal enhances Non-rapid eye movement sleep in pentobarbital-treated mice. CNS Neurosci Ther.

[21] Hosseinzadeh H, Sadeghnia HR (2007). Protective effect of safranal on pentylenetetrazol-induced seizures in the rat: involvement of GABAergic and opioids systems. Phytomedicine.

[22] Hosseinzadeh H, Sadeghnia HR (2005). Safranal, a constituent of Crocus sativus (saffron), attenuated cerebral ischemia induced oxidative damage in rat hippocampus. J Pharm Pharm Sci.

[23] Karimi GR, Hosseinzadeh H, Khalegh PanahP (2001). Study of antidepressant effect of aqueous and ethanolic extract of Crocus sativus in mice. Iran J Basic Med Sic.

[24] Papandreou MA, Tsachaki M, Efthimiopoulos S, Cordopatis P, Lamari FN, Margarity M (2011). Memory enhancing effects of saffron in aged mice are correlated with antioxidant protection. Behav Brain Res.

[25] Hooshmandi Z, Rohani AH, Eidi A, Fatahi Z, Golmanesh L, Sahraei H (2011). Reduction of metabolic and behavioral signs of acute stress in male Wistar rats by saffron water extract and its constituent safranal. Pharm Biol.

[26] Rezaee R, Hosseinzadeh H (2013). Safranal: from an aromatic natural product to a rewarding pharmacological agent. Iran J Basic Med Sci.

[27] Paxions G, Watson C (1988). The Rat Brain.

[28] Schwarcz R, Brush GS, Foster AC, French ED (1984). Seizure activity and lesions after intrahippocampal quinolinic acid injection. Exp Neurol.

[29] Benzie IFF, Strain J (1996). The ferric reducing ability of plasma (FRAP) as a measure of antioxidant power: The FRAP assay. Anal Biochem.

[30] Ellman G (1959). Tissue sulfhydryl groups. Arch Biochem Biophys.

[31] Buege JA, Aust SD (1978). Microsomal lipid peroxidation. Method Enzymol.

[32] Silva JP, Areias FM, Proença FM, Coutinho OP (2006). Oxidative stress protection by newly synthesized nitrogen compounds with pharmacological potential. Life Sci.

[33] Hosseinzadeh H, Sadeghnia HR (2007). Effect of safranal, a constituent of Crocus sativus (saffron), on methyl methanesulfonate (MMS)-induced DNA damage in mouse organs: an alkaline single-cell gel electrophoresis (comet) assay. DNA Cell Biol.

[34] da RochaMD, Viegas FP, Campos HC, Nicastro PC, Fossaluzza PC, Fraga CA (2011). The role of natural products in the discovery of new drug candidates for the treatment of neurodegenerative disorders II: Alzheimer's disease. CNS Neurol Disord Drug Targets.

[35] Colle D, Hartwig JM, Soares FA, Farina M (2012). Probucol modulates oxidative stress and excitotoxicity in Huntington's disease models in vitro. Brain Res Bull.

[36] Kalonia H, Mishra J, Kumar A (2012). Targeting neuro-inflammatory cytokines and oxidative Stress by minocycline attenuates quinolinic-acid-induced Huntington's disease-like symptoms in rats. Neurotox Res.

[37] Leipnitz G, Schumacher C, Scussiato K, Dalcin KB, Wannmacher CM, Wyse ATD (2005). Quinolinic acid reduces the antioxidant defenses in cerebral cortex of young rats. Int J Dev Neurosci.

[38] Dizdaroglu M, Jaruga P (2012). Mechanisms of free radical-induced damage to DNA. Free Radic Res.

[39] Reynolds A, Laurie C, Mosley RL, Gendelman HE (2007). Oxidative stress and the pathogenesis of neurodegenerative disorders. Int Rev Neurobiol.

[40] Ganzella M, Jardim FM, Boeck CR, Vendite D (2006). Time course of oxidative events in the hippocampus following intracerebroventricular infusion of quinolinic acid in mice. Neurosci Res.

[41] Pláteník J, Stopka P, Vejrazka M, Stípek S (2001). Quinolinic acid-iron(ii) complexes: slow autoxidation, but enhanced hydroxyl radical production in the Fenton reaction. Free Radic Res.

[42] Halliwell B (2006). Oxidative stress and neurodegeneration: where are we now?. J Neurochem.

[43] Hariri AT, Moallem SA, Mahmoudi M, Hosseinzadeh H (2011). The effect of crocin and safranal, constituents of saffron, against subacute effect of diazinon on hematological and genotoxicity indices in rats. Phytomedicine.

[44] Bharti S, Golechha M, Kumari S, Siddiqui KM, Arya DS (2011). Akt/GSK-3β/eNOS phosphorylation arbitrates safranal-induced myocardial protection against ischemia-reperfusion injury in rats. Eur J Nutr.

[45] Hosseinzadeh H, Sadeghnia HR, Rahimi A (2008). Effect of safranal on extracellular hippocampal levels of glutamate and aspartate during kainic acid treatment in anesthetized rats. Planta Med.

[46] Brickell KL, Nicholson LFB, Waldvogel HJ, Faull RLM (1999). Chemical and anatomical changes in the striatum and substantia nigra following quinolinic acid lesions in the striatum of the rat: a detailed time course of the cellular and GABAA receptor changes. J Chem Neuroanat.

[47] Santamaría A, Salvatierra-Sánchez R, Vázquez-Román B, Santiago-López D, Villeda-Hernández J, Galván-Arzate S (2003). Protective effects of the antioxidant selenium on quinolinic acid-induced neurotoxicity in rats: in vitro and in vivo studies. J Neurochem.

[48] Ryu JK, Choi HB, McLarnona JG (2005). Peripheral benzodiazepine receptor ligand PK11195 reduces microglial activation and neuronal death in quinolinic acid-injected rat striatum. Neurobiol Disease.

[49] Ganzella M, Faraco RB, Almeida RF, Fernandes VF, Souza DO (2011). Intracerebroventricular administration of inosine is anticonvulsant against quinolinic acid-induced seizures in mice: An effect independent of benzodiazepine and adenosine receptors. Pharmacol Biochem Behavior.

[50] Sadeghnia HR, Cortez MA, Liu D, Hosseinzadeh H, Snead OC (2008). Antiabsence effects of safranal in acute experimental seizure models: EEG and autoradiography. J Pharm Pharm Sci.

[51] Iadecola C, Anrather J (2011). The immunology of stroke: from mechanisms to translation. Nat Med.

[52] Rogers J (1995). Inﬂammation as a pathogenic mechanism in Alzheimer’s disease. Drug Res.

[53] Braidy N, Grant R, Adams S, Brew BJ, Guillemin GJ (2009). Mechanism for quinolinic acid cytotoxicity in human astrocytes and neurons. Neurotox Res.

